# Tendon Healing Response Is Dependent on Epithelial–Mesenchymal–Tendon Transition State of Amniotic Epithelial Stem Cells

**DOI:** 10.3390/biomedicines10051177

**Published:** 2022-05-19

**Authors:** Valentina Russo, Annunziata Mauro, Alessia Peserico, Oriana Di Giacinto, Mohammad El Khatib, Maria Rita Citeroni, Emanuela Rossi, Angelo Canciello, Eleonora Mazzotti, Barbara Barboni

**Affiliations:** 1 Unit of Basic and Applied Sciences, Faculty of Biosciences and Agro-Food and Environmental Technologies, University of Teramo, 64100 Teramo, Italy; amauro@unite.it (A.M.); apeserico@unite.it (A.P.); odigiacinto@unite.it (O.D.G.); melkhatib@unite.it (M.E.K.); mrciteroni@unite.it (M.R.C.); acanciello@unite.it (A.C.); emazzotti@unite.it (E.M.); bbarboni@unite.it (B.B.); 2 Istituto Zooprofilattico Sperimentale dell’Abruzzo e del Molise “Giuseppe Caporale”, 64100 Teramo, Italy; e.rossi@izs.it

**Keywords:** Achilles tendon, amniotic epithelial stem cells, epithelial–mesenchymal transition, immunomodulation, regenerative medicine, teno-differentiation, tendon regeneration

## Abstract

Tendinopathies are at the frontier of advanced responses to health challenges and sectoral policy targets. Cell-based therapy holds great promise for tendon disorder resolution. To verify the role of stepwise trans-differentiation of amniotic epithelial stem cells (AECs) in tendon regeneration, in the present research three different AEC subsets displaying an epithelial (eAECs), mesenchymal (mAECs), and tendon-like (tdAECs) phenotype were allotransplanted in a validated experimental sheep Achilles tendon injury model. Tissue healing was analyzed adopting a comparative approach at two early healing endpoints (14 and 28 days). All three subsets of transplanted cells were able to accelerate regeneration: mAECs with a lesser extent than eAECs and tdAECs as indicated in the summary of the total histological scores (TSH), where at day 28 eAECs and tdAECs had better significant scores with respect to mAEC-treated tendons (*p* < 0.0001). In addition, the immunomodulatory response at day 14 showed in eAEC-transplanted tendons an upregulation of pro-regenerative M2 macrophages with respect to mAECs and tdAECs (*p* < 0.0001). In addition, in all allotransplanted tendons there was a favorable IL10/IL12 compared to CTR (*p* < 0.001). The eAECs and tdAECs displayed two different underlying regenerative mechanisms in the tendon. The eAECs positively influenced regeneration mainly through their greater ability to convey in the host tissue the shift from pro-inflammatory to pro-regenerative responses, leading to an ordered extracellular matrix (ECM) deposition and blood vessel remodeling. On the other hand, the transplantation of tdAECs acted mainly on the proliferative phase by impacting the density of ECM and by supporting a prompt recovery, inducing a low cellularity and angle alignment of the host cell compartment. These results support the idea that AECs lay the groundwork for production of different cell phenotypes that can orient tendon regeneration through a crosstalk with the host tissue. In particular, the obtained evidence suggests that eAECs are a practicable and efficient strategy for the treatment of acute tendinopathies, thus reinforcing the grounds to move their use towards clinical practice.

## 1. Introduction

Amniotic epithelial stem cells (AECs), derived from placental tissues, have gained considerable attention over the past 20 years in the field of regenerative medicine. They possess elevated proliferation and differentiation capabilities and immunomodulatory properties preserved across species [1,2]. Compared with other types of stem cells, AECs have distinctive advantages, including easy isolation, plentiful numbers, the obviation of ethical debates, and non-immunogenic and non-tumorigenic properties [2,3,4].

Furthermore, AECs possess differences in differentiation potential, secretory function and angiogenic and immunomodulatory activity under different culture conditions that modulate their native biological properties and secreted bioactive molecules producing specific effects depending on their application [1]. In this context, it has been demonstrated that AECs, when co-cultured with fetal tendon explants that release inductive tenogenic soluble factors [5], undergo to a stepwise teno-differentiation and form 3D tendon-like structures [6]. Moreover, an in vitro hypoxic condition enables their epithelial to mesenchymal transition (EMT) [7], supporting their differentiation capacity towards the tenogenic lineage [7]. Moreover, during in vitro expansion AECs undergo EMT [8]. Therefore, researchers have tried to explore different culture methods to maintain stemness and avoid EMT occurrence. Canciello et al. [9] have demonstrated that AECs’ EMT is avoided by adopting standardized culture protocols for in vitro amplification with the use of progesterone (P_4_), which also preserves their native key phenotypic and functional attitude such as stemness, plasticity, and immunomodulatory activity [9]. In particular, P_4_ exerts a powerful inhibitory role on AECs’ mesenchymal transition by interfering with the TGF-β1 signaling pathways [9,10]. Furthermore, P_4_-enhanced basal and LPS-induced AECs increase the anti-inflammatory and decrease the pro-inflammatory expression of cytokines [9].

AECs’ immunomodulatory properties have laid the foundation for the use of these cells in treating inflammatory and immune-based pathologies, and encouraging results have been obtained in different disease models, as tendinopathies that have been associated with a state of persisting inflammation [11,12]. Key inflammatory mediators—such as cytokines, nitric oxide, prostaglandins, and lipoxins—play crucial parts in dysregulating the extracellular matrix (ECM) within tendinopathy [11]. In tendons, during spontaneous healing, repairing and remodeling may take several months [13], and their poor intrinsic regenerative potential leads to a repaired tissue that is consistently different from the native one [14]. Current tendinopathy therapeutic strategies foresee the use of conservative approaches or surgical repair using autografts, allografts, and xenografts, which have a limited success [15]. Recently, stem-cell-based therapy for tendinopathies has been proposed as a promising alternative [16].

Many factors affect the repairing process after tendon injury, amongst which is the orchestration of events occurring during the early phase of its healing which are essential for ECM remodeling and inflammatory modulation. AECs have shown a high tenogenic attitude and may represent an alternative strategy to overcome the unsatisfactory results of conventional treatments in tendon regeneration. Encouraging evidence has previously shown that animal and human AECs (hAECs) are able to stimulate the early tendon healing phase by modulating the inflammatory environment, inducing a shift from pro-inflammatory and pro-fibrotic to pro-regenerative cellular responses, leading to a reduced infiltration of inflammatory cells and ordered deposition of ECM components [17,18,19,20]. However, the direct role of AECs in orchestrating ECM remodeling has also been demonstrated. In fact, engrafted ovine AECs (oAECs) and hAECs within the host tissue have the exceptional ability to in situ stepwise trans-differentiate towards the tendon tissue lineage either in allotransplantation [18] or in xenotransplantation settings [17,18,19,20,21].

According to the culture conditions, AECs can maintain their epithelial phenotype (eAECs) [9], undergo EMT (mAECs) [9], or teno-differentiate (tdAECs) [6,7], which resemble the tendon differentiation stepwise process occurring in vivo (epithelial–mesenchyme teno-differentiation). Thus, this study aims to verify which of these three cell subsets, according to their biological and functional characteristics, is the most effective for tendon regeneration during the early phases of tendon healing.

To this purpose, in this research, the phenotype, genotype, and immunomodulatory properties of the three subsets of AECs were assessed before allotransplanting them in an in vivo validated high translational sheep model for human tendon function/dysfunction [22]. The tendon regenerative influences of the three different AEC phenotypes (i.e., eAECs, mAECs, and tdAECs) were verified on tendon explants by integrating biochemical (gene and protein expression), cellular (in terms of number of cells and cell alignment), morphological (tissue microarchitecture recovery and ECM organization), and immunomodulatory (expression of M1 and M2 Mφ markers, IL10 and IL12 and blood vessels) outcomes during the early phase of tissue healing (14 and 28 days) in order to verify which of the used cell subsets is the most suitable candidate for its translation in treatment of tendon injuries.

## 2. Materials and Methods

### 2.1. Ethics Statement

Ovine AECs and fetal tendon explants (FT), used within the study, were obtained at the local slaughterhouse from discarded tissues (fetuses and amniotic membranes of pregnant slaughtered animals) of feed chain sheep. For this reason, for these samples no ethics statements are required.

Sheep Achilles tendon experiments were conducted in compliance with the Italian National Laws (Legislative Decree n.26/2014) and with the European Community Council Directive 2010/63/EU on the Protection of Animals used for Scientific Purposes, upon approval by the Ministry of Health (approval ID 1205/2015-PR of 18 November 2015). The sheep were bred according to E.D. 2010/63/UE before performing Achilles tendon lesions. Animals were quarantined for 2 weeks to check the general healthy status. Surgical procedures were carried out in an authorized veterinary hospital.

### 2.2. AEC Isolation and Culture

AECs were obtained by isolation from ovine amniotic membranes (AM) from 3 different fetuses of 25–35 cm of length at ∼2–3 months of pregnancy [23] as previously described [6], and treated with different culture conditions to reach a specific cell’s phenotype and genotype prior to implantation into induced tendon defect. To obtain cells for the implantation, AECs were seeded in standard medium (SM) [7], without any further treatment to obtain mesenchymal AECs (mAECs) or in SM with 25 μM of P_4_ to preserve epithelial AECs’ (eAECs) original phenotype until 3 passages, both in air incubator with 5% CO_2_ at 38 °C [9,18]. To obtain tenogenic differentiated AECs (tdAECs) for the implantation, freshly isolated AECs from the AM were cultured in a co-culture system with FT as described in previous articles [6,7]. For the further experiments and transplantation only the tdAECs obtained from the formed 3D tendon-like structures were used. In detail, the 3D tendon-like structures were isolated manually under the stereomicroscope and trypsinized with 0.25% trypsin EDTA (Sigma Aldrich, St. Louis, MO, USA) to separate the cells.

Before cell transplantation, all cell typologies (mAECs, eAECs, tdAECs) were subjected to fluorescent cell membrane labelling with PKH26 (S-MINI26-1KT, Sigma Aldrich, St. Louis, MO, USA). In detail, PKH26 linker dye stably incorporates into lipid regions of the cell membrane. Due to this extremely stable fluorescence, PKH26 is the linker dye of choice for in vivo cell tracking and monitoring studies [18] (https://www.sigmaaldrich.com/specification-sheets/393/296/MINI26-BULK.pdf, accessed on 5 January 2022).

Briefly, the different subsets of AECs were re-suspended in 1 mL of Diluents C and then added at 1 mL of Dye Solution containing 4 µL of PKH26. The cellular suspension was incubated for 5 min at room temperature with periodic mixing. Cell staining was stopped with 2 mL of 1% BSA in PBS for 1 min and finally centrifuged at 400× *g* for 10 min. Cells were suspended and counted in order to obtain 1 × 10^7^ PKH26-marked vital cells to be used for transplantation. Cells were overnight preconditioned with homologous sera derived from the animals enrolled for this study. Aliquots of 1 × 10^7^ of differently obtained AEC typologies were stored in liquid nitrogen in vials until their transplantation.

### 2.3. AEC In Vitro Genotype Characterization

The epithelial, EMT, and tendon-related markers were evaluated by analyzing the expression of the related genes as for the EMT *Cytokeratin 8* (*CYTO8*) and *Vimentin* (*VIM*) and for tendon differentiation *Scleraxis B* (*SCXB*), *Collagen Type 1* (*COL1*), and *Tenomodulin* (*TNMD*) on eAECs, mAECs, and tdAECs as previously described [7]. The values were normalized to endogenous reference gene GAPDH [7,17,19]. The relative expression of different amplicons was calculated by the comparative Ct (ΔCt) method, converted to relative expression ratio (2^−ΔΔCt^) [24] and expressed as fold change over freshly isolated AECs (T_0_) = 1. The reaction of RT-PCR was performed in triplicate for each experimental replicate of each different biological sample (*n* = 3). The gene primers were designed using Primer3 and BLAST from NIH, and details are specified in Table 1.

### 2.4. Phenotype Assessment of the Different AEC Experimental Groups

To characterize the phenotype of each typology of in vitro cultured AECs (mAECs, eAECs, tdAECs), the protein expression and localization of CYTO8, VIM, and TNMD were recorded by conducting immunocytochemistry (ICC) analyses following standardized protocols [7,9]. Freshly isolated AECs (T_0_) were used as a control. The omission of primary antibodies (Abs) was used as negative control. Details on Abs and dilutions are specified in Table 2. The images were evaluated using a Zeiss Axioskop 2 Plus incident light fluorescence microscope (Carl Zeiss, Oberkochen, Germany), which was equipped with a CCD camera (Axiovision Cam, Carl Zeiss) with a resolution of 1300 × 1030 pixels, configured for fluorescence microscopy, and interfaced to a computer workstation with an interactive and automatic image analyzer (Axiovision, Carl Zeiss) [25]. The reactions were carried out in triplicate on each biological replicate (*n* = 3) for each experimental condition.

### 2.5. Comparison of In Vitro Immunomodulatory Properties of eAECs, mAECs vs. tdAECs

The immunomodulatory activities of eAECs, mAECs, and tdAECs were analyzed on peripheral blood mononuclear cells (PBMCs) by testing their proliferation modifying a previously reported protocol [26]. Briefly, ovine PBMCs were obtained by density gradient centrifugation (Lymphoprep, Axis-Shield, Oslo, Norway) of 15 mL peripheral blood, as previously described [27]. Lymphocyte proliferation was obtained by addition of phytohemagglutinin (PHA; Sigma Aldrich, St. Louis, MO, USA) at a final concentration of 2 μg/mL. Different concentrations of eAECs, mAECs, and tdAECs (2 × 10^5^, 1 × 10^5^, 0.5 × 10^5^, 0.25  ×  10^5^, and 0.125  ×  10^5^) were cultured in cell-to-cell contact setting in RPMI complete medium and left to adhere overnight. The next day, eAECs, mAECs, and tdAECs were γ-irradiated (4000 cGy) and 2  ×  10^5^ PBMCs/PHA was added to each well with or without 1 µg/mL LPS for 24 h, obtaining PBMC:AEC ratios of 1:1, 1:0.5, 1:0.25, 1:0.125. Lymphocyte proliferation was assessed after 3 days of culture by adding 0.67 μCi per well of [3H]-thymidine (MP Biomedicals^TM^ Inc., Irvine, CA, USA) for 16–18 h. Cells were then harvested with a Filtermate Harvester (PerkinElmer, Shelton, CT, USA), and thymidine incorporation was measured using a microplate scintillation and luminescence counter (Top Count NXT; PerkinElmer, Shelton, CT, USA).

### 2.6. Ovine Achilles Tendon Injury Model

**Figure 1:**
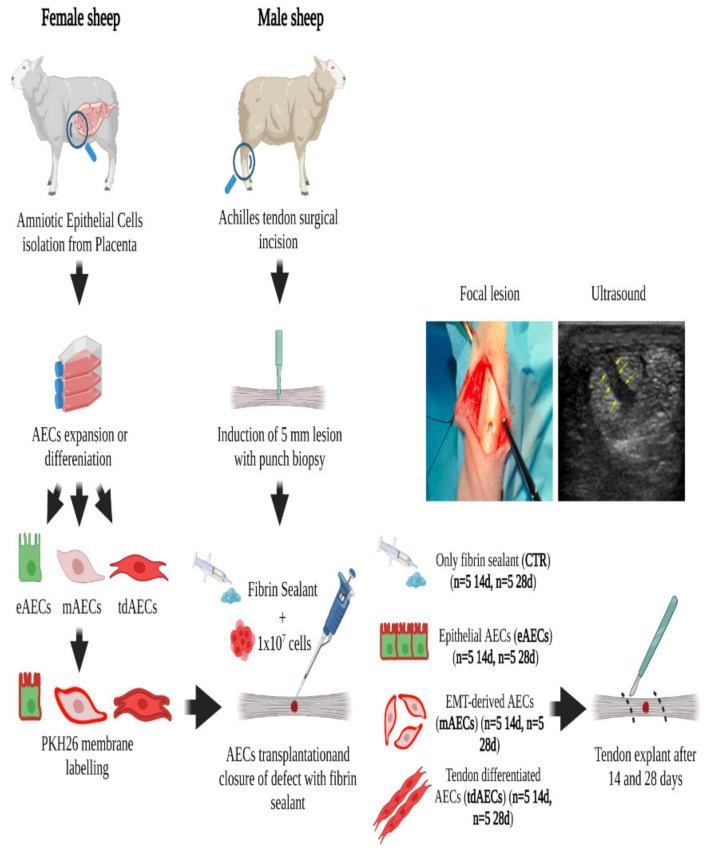
Animal experimental design. Workflow of the animal experimental design in which different subsets of oAECs (eAECs, mAECs, tdAECs) were allotransplanted into a validated sheep Achilles tendon injury model. In the Figure, a representative image of the focal lesion carried out with the biopsy punch and the corresponding US image showing the hypoechoic area corresponding to the lesion within the tendon (yellow arrows).

Forty adult male sheep (2 years old), with an average weight of 40 ± 6 kg, were bred in an authorized farm. Only male animals were used for these studies instead of female animals to avoid any possible physiological P_4_ influence. The animal experimental design is summarized in Figure 1.

At the time of surgery, anesthesia was induced by administering xylazine IM (Rompun^®^ 0.2 mg/kg; Bayer HealthCare, Leverkusen, Germany) and tiletamine-zolazepam IV (Zoletil 100 0.2 mg/Kg; VIRBAC S.r.l., Milan, Italy). The intubated sheep were kept under general anesthesia by inhaling 2.5% Halothane^®^ (Merial Italia S.p.A., Milan, Italy) in an oxygen mixture. The pelvic limbs of the animals were placed off with both tarsi under flexion. A 3 cm skin incision was made, starting at 4 cm proximal to the tuber calcis. The medial and more prominent component of Achilles tendon, that is, the tendon of m. flexor digitorum superficialis, was isolated. Using a sterile punch, a full thickness hole of 5 mm in diameter was performed only on the left Achilles tendons. The defect of each limb used as control (CTR) was filled only with fibrin glue (60 µL, 1:1, *v/v*; Tissucol/DMEM; Baxter S.p.A., Illinois, IL, USA), whereas the treated tendon defects were filled with 1 × 10^7^ of different AECs’ typologies: eAECs, mAECs, or tdAECs, obtained from in vitro cultured AECs under different conditions, previously stained with PKH26 dye as previously described and then sealed with fibrin glue [17]. In particular, five animals were employed for each time point (14 and 28 days) and each cell subset (eAECs, mAECs, and tdAECs) and CTR.

The paratenon and fascia were closed before skin suture. The wounds were weekly inspected with a Toshiba Nemio 20 (Toshiba Medical Systems Corporation, Otawara, Japan) ultrasound (US) equipped with a linear probe at 7.5 MHz (multi-frequency 6/12 MHz) in order to verify the effectiveness of the experimental lesion and tendon regeneration until sacrifice. After surgery, animals were kept in a small sheepfold until sacrifice. Animals were euthanized at 14 and 28 days after surgery by overdose of thiopental (Pentothal Sodium-Intervet) and embutramide (Tanax^®^-Intervet, Aprilia, Italy).

### 2.7. Gene Profiles of the Healing Tendons after Explant

#### 2.7.1. Laser Capture Microdissection (LCM) Technique

The explanted tendons, 14 and 28 days after transplantation, were transversally cut at least 5 mm from the injured area according to previously published reports [17,18,19]. The specimens were frozen in liquid nitrogen. Cryosections 7 μm in thickness were briefly air-dried on uncoated glass slides and washed with 70% ethanol. The sections were kept on dry ice at −80 °C until they were subjected to LCM. Just before the procedure, the sections were fixed in 70% ethanol for 10 s and stained with H&E according to Hoffman et al. [28]. LCM was performed by using a laser capture microdissection (MMI Cellcut device, Eching, Germany) apparatus. The settings of the laser were performed as follows: spot diameter 10 µm, pulse duration 50 ms, laser power 50 mW. The area to be micro dissected was identified under a light microscope at ×640 magnifications. The micro dissected injured area including the implantation site was dropped onto a separate cap before going on to total RNA extraction. The area of healthy tendon contiguous to the injured area of each animal was also considered for microdissection in order to collect healthy tendon cryosection as internal control of each animal for the investigations. Total RNA from all microdissected sections was extracted and used RT-qPCR procedures as described below.

#### 2.7.2. Total RNA Extraction and RT-qPCR for Tendon Explants

The tenogenic differentiation: *SCXB, Collagen type 3* (*COL3*), *COL1*, *TNMD*, and *tromphospondin* (*THBS4*), the pro-inflammatory (*CD86* and *IL12*) and anti-inflammatory (*CD206* and *IL10*) genes (Table 1) were evaluated by RT-qPCR on eAECs, mAECs, treated tdAECs, and CTR tendons’ cryosections. Healthy tendons of contiguous injured area/animal and contralateral healthy tendon/animal were used to evaluate the baseline gene expressions. Total RNA was extracted from tendon microdissected defect area (*n* = 30 for each animal group/time) and from healthy tendon microdissected contiguous to the injured area cryosections (*n* = 30 for each animal group/time) by TriReagent (Sigma Aldrich, St. Louis, MO, USA) following manufacturer’s instruction as previously reported [17,19]. After evaluation of RNA integrity and DNaseI digestion 1 μg of total RNA of each sample was used for reverse transcription reaction in cDNA. Two-step cycling RT-qPCR analysis was performed, as previously described [7], by using the specific tenogenic and immunomodulatory gene primers (Table 1). Each gene value was normalized to endogenous reference gene GAPDH. For each treated animal, the intra-relative expression of each target gene in injured tendon was calculated by the comparative Ct (ΔΔCt) method [24] to contiguous and contralateral healthy tendon. In order to compare the gene expression between different treated animal groups, the target gene values were expressed as fold change over CTR tendon set as 1. For statistical analyses, the mean of three independent experiments/animal was considered.

### 2.8. Protein Profiles of the Healing Tendons after Explant

#### 2.8.1. ECM Analysis of Tendon Explants: Histology and Immunohistochemistry

The explanted tendons, 14 and 28 days after transplantation, were transversally cut at least 5 mm from the injured area and placed in liquid nitrogen. Cryosections, 7 μm in thickness, were processed with H&E and immunohistochemistry (IHC).

The H&E was performed as previously reported [17] to collect overall information on the microarchitecture of the tendon within the injured districts during the different interval points (14 and 28 days).

IHC analyses were performed with the antibodies summarized in Table 2 following previously published protocols [17,18,19].

The fluorescence intensities of the analyzed tendon samples immunostained for COL3, COL1, DCN, and TNMD were assessed through RGB profiler plugin of ImageJ software (NIH). Each captured image was processed through this plugin which draws the red, green, and blue profile plot of an RGB image on the same plot, for each type of line selection. The analyzed area is represented as average fluorescence intensity and visualizing both the minimum and maximum fluorescence intensity. Two reference values were set for each analyzed protein: to determine maximum fluorescence intensities the healthy tendon was used (for COL3 the endotenon), instead, the minimum fluorescence intensities were determined from the background of the analyzed areas. At least three images of each analyzed area belonging to all tested groups were used.

The PKH26 (λ_excitation_ = 551 nm, λ_emission_ = 567 nm) labelled AECs were retrieved with an Axioskop 2plus microscope (Zeiss). Sections with retrieved PKH26-positive cells were immunostained for COL1 and TNMD as described above.

#### 2.8.2. Histomorphometric Analyses on Tendon Explants

Morphometric analyses were conducted on the IHC acquired images and were performed by using guided programs to count:

- Cellularity;
- Cell alignment;
- CD86- (M1 macrophages) and CD206-positive (M2 macrophages) cells.

All morphometric analyses were performed at ×200 magnification by acquiring 5 randomly selected fields from 6 contiguous areas starting from the healthy one (area 0) and continuing throughout the repairing areas (from area 1 to area 5), where area 5 represented the core of the lesion. The extension of each analyzed area was 500 µm and, therefore, the total area analyzed within each section was 30,000 µm^2^ [17].

Cellularity was quantified on the total number of DAPI-stained nuclei in each acquired field of each treated group determined at 14 and 28 days through ImageJ software (NIH image).

Cell alignment was represented by the orientation of the cells with respect to the longitudinal axis of the harvested tendons of the different treated groups at 14 and 28 days. This parameter was assessed using the Directionality Plugin of ImageJ [7,25,29,30] to better determine the teno-inductive potential of the different treatments on area 1 (near healthy area) and area 4 (near the core of the lesion, area 5). Briefly, the IHC images used to determine cellularity were chopped by the Plugin into square pieces on which it was computed the Fourier power spectra allowing the generation of statistics data. The elaborated data are expressed as direction and dispersion, which represent the highest peak found at the center of the Gaussian and the standard deviation (S.D.) of the Gaussian, respectively. When the cells were oriented along the longitudinal axis of the sample, the direction value was equal to 0. The increase in dispersion values meant that the homogeneity of cell orientation was low. Afterwards, the direction values were normalized to area 0 which refers to the healthy tendon. Indeed, the normalized data to healthy tendons were expressed as angle deviation.

Pro-inflammatory M1Mφ phenotype population (% CD86-positive cells/total number of nuclei in the area), and anti-inflammatory M2Mφ phenotype populations (% CD206-positive cells/total number of nuclei in the area) were quantified.

Analyses were carried out on at least 5 different sections of analyzed tendon specimen/treated group.

Moreover, cellularity, cell alignment, extracellular matrix COL1 fiber deposition, and blood vessel organization were combined to generate four different scores to define the regeneration of the tendon microarchitecture as shown in Table 3, by reporting some modifications to previously published works [17,18].

### 2.9. Statistical Analysis

The quantitative data of the AECs’ in vitro characterization were assessed for their normality distribution with D’Agostino Pearson test, and then they were compared as mean ± S.D. by using One-Way ANOVA followed by Tukey post hoc test (GraphPad Prism 9, GraphPad Software, San Diego, CA, USA) on at least three samples for each experimental condition performed in triplicate on each biological replicate (*n* = 3 animals). Significant values were considered at least for *p* < 0.05.

The quantitative data of the in vivo experiments were assessed for their normality distribution by D’Agostino Pearson and expressed as mean ± S.D. by using One-Way ANOVA followed by Tukey post hoc test (GraphPad Prism 9, GraphPad Software, San Diego, CA, USA) on all tested animals belonging to CTR and stem-cell-treated groups, conducted in triplicates.

## 3. Results

### 3.1. In Vitro Production of Epithelial, Mesenchymal, and Tendon-like Committed Typologies of AECs

Ovine AECs were evaluated to confirm their genotype and phenotype before transplantation when amplified with P_4_ (eAECs), or to undergo EMT during in vitro expansion (after three passages; mAECs), or to differentiate into tendon-lineage-derived cells over 14 days of co-culture with FT (tdAECs).

**Figure 2:**
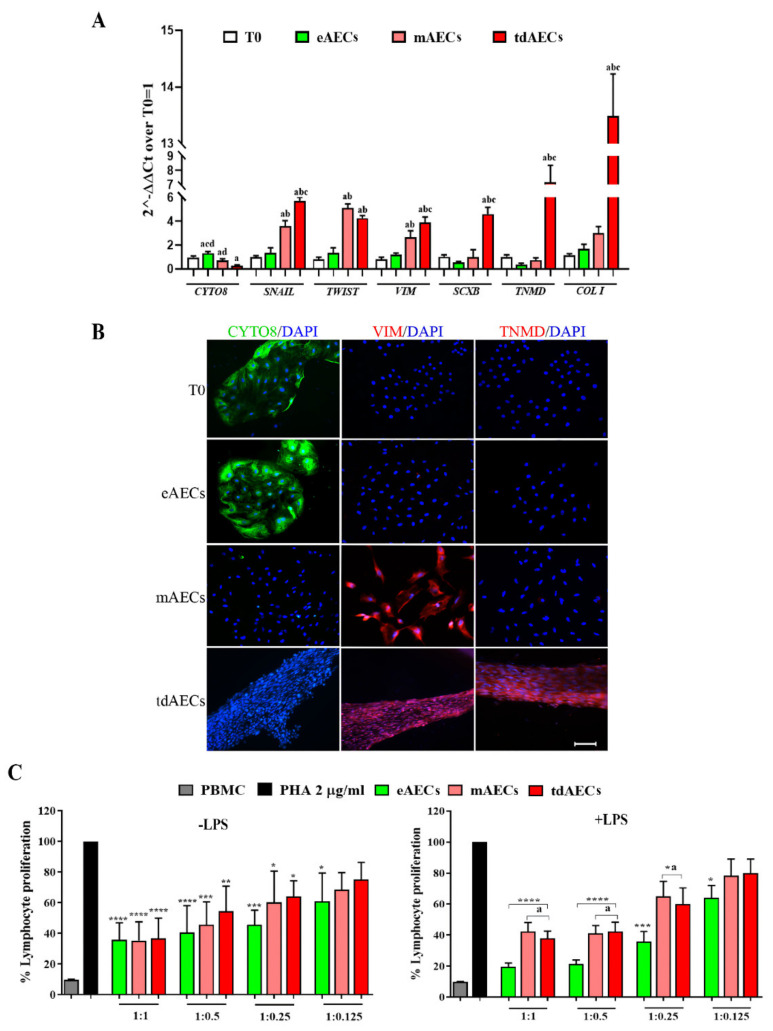
Characterization of oAECs under different culture conditions. (**A**) Gene expression profile assessed by real-time qPCR analysis of EMT (*CYTO8*, *SNAIL*, *TWIST,* and *VIM*) and tendon-related genes (*SCXB*, *TNMD*, *COL 1*) in mAECs, eAECs, and tdAECs. Values were considered significant for *p* < 0.05 and expressed with the superscript: ^a^ vs. T_0_, ^b^ vs. eAECs, ^c^ vs. mAECs, ^d^ vs. tdAECs. The quantitative data were expressed as mean ± S.D. (**B**) Representative ICC images of EMT and the tendon-related marker TNMD. ICC was assessed to detect the presence of CYTO8 (green fluorescence), VIM (red fluorescence), and TNMD (red fluorescence) protein in eAECs, mAECs, and tdAECs. Nuclei were counterstained with DAPI (blue fluorescence). Scale bar = 50 µm. (**C**) Immunomodulatory effect of eAECs, mAECs, and tdAECs on PBMCs’ proliferation in absence or presence of LPS stimulus of in vitro culture by cell contact. The different subsets of oAECs were added to obtain different PBMC:oAEC ratios (1:1, 1:0.5, 1:0.25, 1:0.125). Data are represented as mean ± S.D. Values of independent experiments performed in triplicate. Statistical values were considered differently significant for * *p* < 0.05, ** *p* < 0.01, *** *p* < 0.001, and **** *p* < 0.0001 vs. PHA-stimulated PBMCs (in absence of the different subsets of oAECs) and ^a^ vs. eAECs. The quantitative data were expressed as mean ± S.D.

The analyses of genotypic (Figure 2A) and phenotypic (Figure 2B) profiles confirm that AECs were able to preserve in the presence of P_4_ the native epithelial cobblestone shape (eAECs) by expressing high levels of *CYTO8* (*p* < 0.001 vs. T_0_) and displaying a widespread positivity for this epithelial protein marker. At the same time, named eAECs did not contain the late mesenchymal marker *VIM* and showed a basal expression of both early (*SCXB*) and late (*TNMD* and *COL1*) tendon-related genes.

On the contrary, AECs amplified in the absence of any stimulus were accompanied by a spontaneous in vitro activation of EMT. At the end of the third passage of expansion, the majority of the cells acquired a mesenchymal phenotype displaying an elongated shape combined with a high intracellular content of VIM and a drastic loss of CYTO8 (*p* < 0.05 vs. T_0_) and the relative proteins. Moreover, these cells, which have been classified as mAECs, upregulated the EMT transcription genes (*SNAIL* and *TWIST p <* 0.05 vs. T_0_ and eAECs), *VIM* and *COL1* (*p* < 0.01 and *p* < 0.05 vs. T_0_ and eAECs, respectively; Figure 2A).

Finally, the third typology of cells named tdAECs at the end of the teno-inductive condition acquired a tendon-like phenotype demonstrated by the persistence of mesenchymal protein profile (VIM positivity and CYTO8 negativity: Figure 2A,B) in combination with a widespread intracellular expression of the late tendon-related marker, TNMD (Figure 2B). Their genome profile confirmed tdAECs’ shift with low levels of *CYTO8* expression, the maintenance of the expression of *SNAIL* and *TWIST,* and the upregulation of *SCXB, TNMD,* and *COL1* tendon-related genes (Figure 2A).

Taken together, these results confirm the acquisition of epithelial, mesenchymal, and tendon-like profiles of the three typologies of cells used for the preclinical studies.

### 3.2. The Three Typologies of AECs Differed for In Vitro Immunomodulatory Properties

The three AEC subsets displayed in vitro a basal and LPS-induced immunomodulatory activity that was, however, strictly dependent on AEC phenotype.

Even if all AEC typologies were able to downregulate proliferation in lymphocytes activated by PHA under a cell-to-cell system, eAECs were able to express a higher immunomodulatory performance in both the assay conditions (basal vs. LPS) (Figure 2C). The analysis of PMBC proliferation data, indeed, showed that the inhibitory effect was in all the subsets of dose-dependent AECs until the ratio 1:0.125 (PMBC:AECs, respectively), when these did not receive any preliminary stimulation (Figure 2C). Under basal condition, the greatest inhibitory influence of eAECs became evident at a 1:0.25 ratio (Figure 2C). Of note, the preliminary LPS priming, which mimics in AECs an inflammatory stimulus, significantly increased the inhibitory effect on PBMCs (Figure 2C). However, eAECs displayed the greatest inhibition (80% of eAECs vs. approximately 65% of mAECs and tdAECs) and retained negative influence (until 1:0.125 ratio) on PBMCs’ proliferation (Figure 2C).

### 3.3. AEC Allotransplantation in Ovine Achilles Injured Tendon

**Figure 3:**
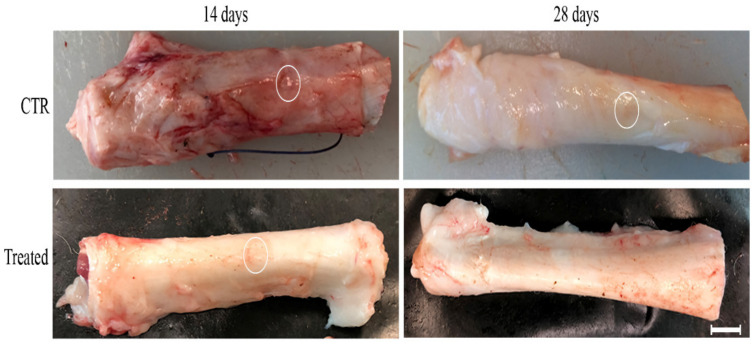
Macroscopic morphology of the explanted ovine Achilles tendons at day 14 and 28. Representative images of healthy, CTR, and treated Achilles tendons explanted after 14- and 28-day treatments. The 5 mm circular defects created in the ovine Achilles tendons are evidenced within the white circle; in treated tendons explanted at 28 days the defect is not distinguishable anymore. CTR tendons were filled with fibrin glue, whereas the treated tendons with 1 × 10^7^ PKH26-stained eAECs, mAECs, or tdAECs cells suspended in fibrin glue. Scale bar = 0.3 cm.

All three cell subsets (i.e., eAECs, mAECs, and tdAECs) were separately allotransplanted in a validated model of mechanical tendon injury (Figure 3). The surgical procedures and cell transplantation did not impact animal behavior, which recovered complete movement immediately after anesthesia. Weekly US follow-up revealed, coherently with previous evidence [17], that under spontaneous healing (CTR) the tissue displayed the lesioned area and evident signs of inflammation during the 28 days. By contrast, the last US carried out at day 28 allowed to document in the majority of transplanted tendons (four out of five eAECs, three out of five mAECs, and in the totality of tdAECs) the first signs of tissue recovery with a more regular longitudinal echogenic pattern inside the injured zone (data not shown).

The totality of CTR explants (five out of five) displayed a hemorrhagic area inside and surrounding the tendon defect at day 14. Furthermore, they presented a diffuse oedema with a diameter larger than the healthy tendons. By contrast, the allotransplanted tendons, independently of the cell subsets used (four out of five in all the three subset typologies of treated tendons), showed a limited swelling already at day 14. Both the hemorrhagic phenomena and oedema decreased also in CTR tissues at day 28 post-surgery, even if the macroscopic swelling in this group of tendons persisted (Figure 3).

### 3.4. AEC Phenotype Subsets Strongly Impact the Expression of Tendon-Related and ECM Gene Markers

**Figure 4:**
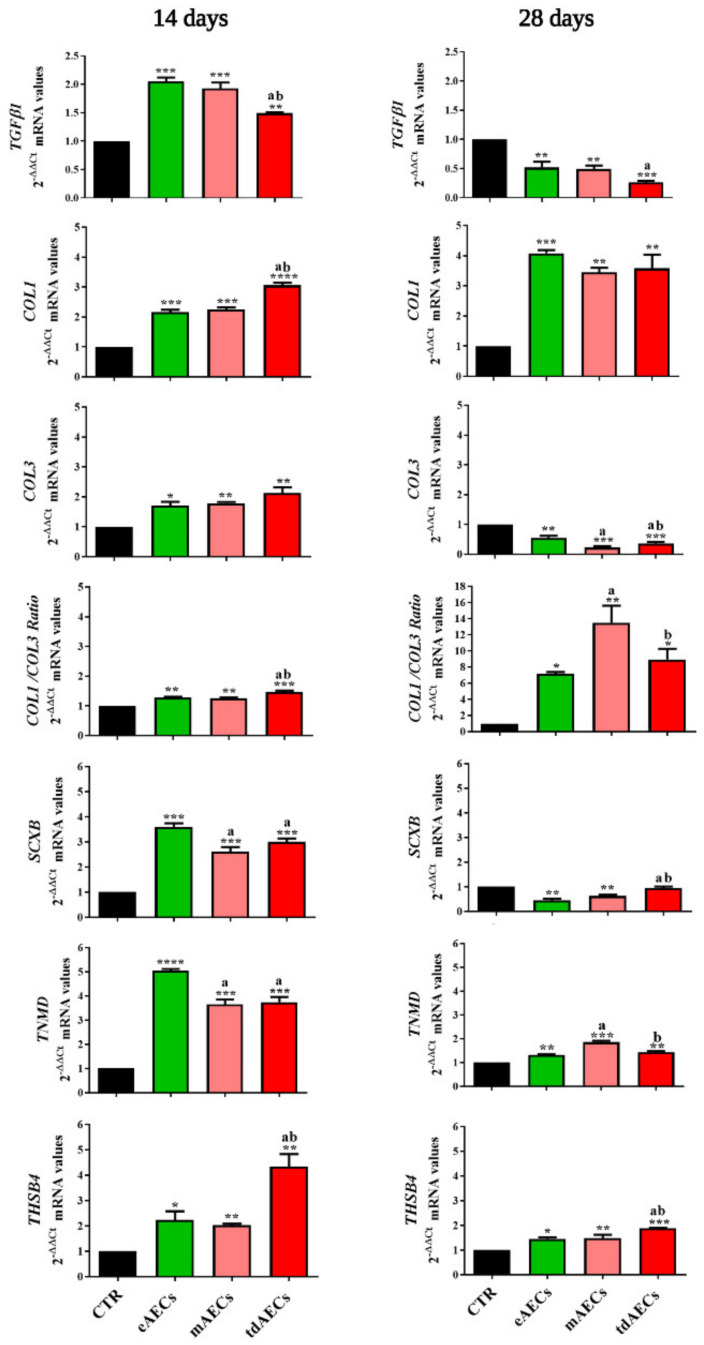
Gene expression profile of ECM and tendon-related genes in explanted tendons. Gene expression profile of *TGFβ1, COL1, COL3, SCXB, TNMD,* and *THSB4* in CTR (black), eAECs (green), mAECs (light red), and tdAECs (red)-treated tendons at 14 and 28 days after cells’ transplantation. Values were considered statistically significant for * *p* < 0.05, ** *p* < 0.01, *** *p* < 0.001, **** *p* < 0.0001 vs. CTR, ^a^ *p* < 0.05 vs. eAECs, ^b^ *p* < 0.05 vs. mAECs. The quantitative data were expressed as mean ± S.D.

In order to evaluate the in vivo processes of ECM remodeling and tendon healing, the profile of the related genes was compared in treated tendons at 14 and 28 days after spontaneous repair or stem cell allotransplantation (Figure 4). In order to document exclusively the kinetic profiles of ECM and tendon-related genes in the injured tissues, the transcripts were isolated from the lesioned area of the tendon with the aid of the LCM technique.

These analyses have pointed out that at day 14 several key genes showed a significant upregulation in the treated tendons such as *TGF-β1*, *COL1*, *COL3*, *SCXB*, *TNMD*, and *THBS4*. However, the amounts of transcripts isolated from allotransplanted tissues were strongly influenced by the different AECs’ typologies (Figure 4). In particular, the ECM-inducing factor *TGF-β1* was significantly upregulated in eAEC and mAEC transplanted tissues (*p* < 0.05 vs. tdAECs and *p* < 0.001 vs. CTR, Figure 4). At the same time, eAECs were able to significantly stimulate the expression of *SCXB* (*p* < 0.001 vs. CTR, *p* < 0.05 mAECs and tdAECs, respectively) and *TNMD* (*p* < 0.0001 vs. CTR, *p* < 0.05 mAECs and tdAECs, respectively) while, conversely, *THSB4* was strongly upregulated in tissues receiving tdAECs (*p* < 0.05 vs. eAECs and mAECs, *p* < 0.001 vs. CTR, Figure 4). Similarly, tdAECs led a significant increase in *COL1* gene expression in the host tissue by determining a more positive early COL1/COL3 ratio (*p* < 0.05 vs. eAECs and mAECs, respectively, and *p* < 0.001 vs. CTR).

The gene pattern profile substantially changed at day 28 inside the injured zone. All oAEC subset transplantations induced an overall reduction in *TGF-β1* and tendon-related gene expression except for *COL1*. Furthermore, the overall analysis showed that most of the key late tendon-related transcripts, such as *TNMD*, *THSB4* and *COL1*, maintained higher levels in transplanted tissues than in CTR ones (Figure 4). Moreover, AEC subset-related modulation of gene expression also persisted at day 28.

More in detail, the downregulation of *TGF-β1* mRNA expression was strongly induced by tdAEC transplantation (*p* < 0.05 vs. eAECs and mAECs, Figure 4). A significant downregulation of *SCXB* was observed in eAEC- and mAEC-treated tendons (*p* < 0.05 vs. tdAECs), of *TNMD* in eAECs and tdAECs (*p* < 0.05 mAECs vs. eAECs and tdAECs, respectively), and of *THBS4* in tdAEC-treated tendons (*p* < 0.05 vs. eAECs and mAECs, Figure 4). This evidence showed that only eAEC transplantation was able to determine a significant reduction in the expression of the major tendon-related genes at day 28.

Similarly, the analysis of the ECM gene expression also supported the idea of a more advanced process in tendon regeneration promoted by all subsets of AEC treatments. Indeed, *COL1* transcripts were significantly higher in explants that received the subsets of AECs than those that healed spontaneously (CTR vs. eAECs, *p* < 0.001 and CTR vs. mAECs or tdAECs, both *p* < 0.01), whereas *COL3* mRNA showed a significantly lower availability (CTR vs. mAECs, *p* < 0.001 and CTR vs. eAECs or tdAECs, for both *p* < 0.01). As a consequence, a greater COL1/COL3 ratio was recorded in all the typologies of allotransplanted tendons (CTR vs. mAECs, *p* < 0.01 and CTR vs. eAECs or tdAECs, for both *p* < 0.05, Figure 4).

### 3.5. AECs’ Allotransplantation Promoted the Early Tendon ECM Healing with Higher Performances in eAEC- and tdAEC-Treated Tissues

**Figure 5:**
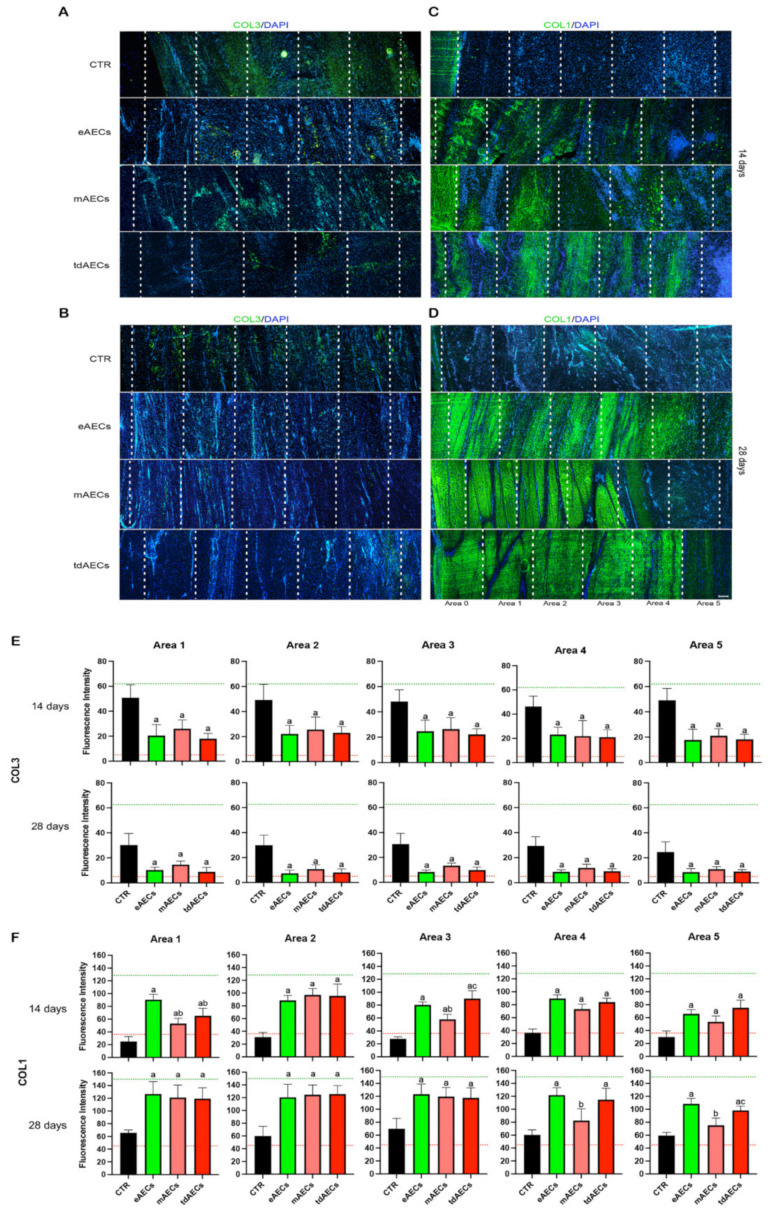
ECM composition after transplantation of eAECs, mAECs, and tdAECs in injured tendons. In each panel the healthy portion (area 0 positioned on the left in all images) and the injured area of CTR and treated explants (from area 1 to area 5) are shown with white dashed lines. COL3 (green fluorescence) representative images at (**A**) 14 days and (**B**) 28 days which was undetectable in healthy tissues (area 0) an barely expressed in eAEC-, mAEC- and tdAEC-treated tendons especially at 28 days, whereas it persisted in CTR tendons at all time points. Representative images of COL1 fibers (green fluorescence) at (**C**) 14 days and (**D**) 28 days which were detected in the healthy portion of the explants (area 0) and within the injury site of eAEC-, mAEC-, and tdAEC-transplanted tendons. Nuclei were counterstained with DAPI. Scale bar = 100 µm. Representative histograms of green fluorescence intensities of (**E**) COL3 and (**F**) COL1 at 14 and 28 days within CTR (black), eAEC- (green), mAEC- (light red), and tdAEC- (red) treated groups in all analyzed areas using RGB profiler plugin of ImageJ software (NIH). The green and red dashed lines represent the maximum and minimum green fluorescence intensity of COL3 and COL1, respectively. ^a^ Statistically significant vs. CTR (*p* < 0.05), ^b^ statistically significant vs. eAECs (*p* < 0.05), ^c^ statistically significant vs. mAECs (*p* < 0.05).

The analysis of the Achilles tendon microarchitecture carried out with H&E (data not shown) and IHC and fluorescence intensity of the main ECM proteins (Figure 5A–F) revealed that all AEC subsets markedly accelerated the early phase of tendon healing even if the process of ECM remodeling was influenced by the stem cell phenotype (eAECs, mAECs, tdAECs) used.

The analysis of tendon explants after 14 days showed that no traces of fibrin glue persisted, as well as that no foci of exogenous mesenchymal-derived tissues were differentiated (bone, cartilage, or adipose tissue).

IHC (Figure 5A–D) and green fluorescence intensity (Figure 5E,F) analyses clearly showed that in CTR tissues COL3, which normally is localized in the paratenon and endotenon of healthy tendons, was widespread in the repairing site at both experimental endpoints (Figure 5A,B,E), whereas COL1 was barely detectable and scattered with a random distribution (Figure 5C,D,F).

On the contrary, the allotransplanted tendons at day 14 showed very low levels of COL3, which was mainly distributed in restricted areas to then become barely detectable at day 28 (Figure 5A,B,E). Indeed, IHC supported the evidence of an accelerated replacement of COL3 with COL1 in all three AEC-transplanted tendons. The stimulatory influence of all subsets of AECs on ECM remodeling led to COL1 fibers to acquire a more mature state of organization after 28 days from injury with the final parallel orientation along the longitudinal axis of the tendon (Figure 5C,D,F), although some differences could be revealed amongst the different types of the transplanted cells. Specifically, by quantifying fluorescence intensity, it was evident that at day 14 COL1 fibers in eAECs displayed a continuous distribution covering from area 1 to area 4, whereas mAECs and tdAECs promoted a less organized spatial COL1 deposition, presenting inside the injured zone alternated areas with higher and fainter matrix density (Figure 5C,F). COL1-related ECM assembling improved after 28 days in all AEC-treated samples. The higher degree of ECM maturity was observed in area 1, where there was a greater density of COL1 and parallel orientation of fibers that decreased moving towards the core of the lesion (area 5) where COL1 deposition was not totally organized yet. Thus, fluorescence intensity analysis confirmed in all AEC treatments a spatial centrifugal gradient of COL1 remodeling moving from area 0 that represents the healthy portion of tendon towards area 5, the core of the lesion (Figure 5D,F). Of note, eAECs and tdAECs induced a greater extension in COL1 deposition and fiber organization: in four out of five tissue explants analyzed, a mature COL1 ECM was observed from area 1 to 4, differently from mAEC-treated tissue where only one out of five showed this high degree of ECM remodeling, whereas in the remaining tissues (four out of five) this COL1 deposition did not exceed area 3 (Figure 5D,F).

**Figure 6:**
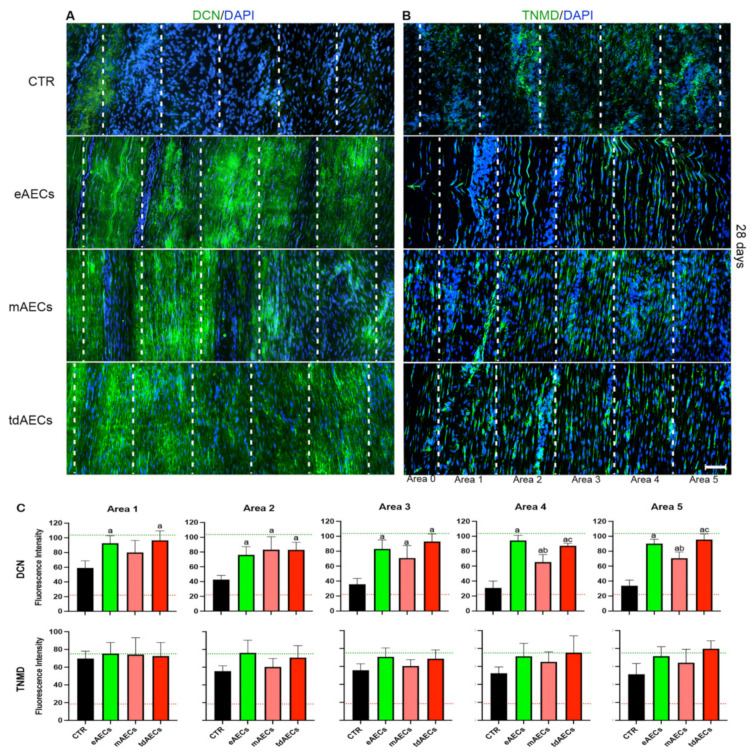
DCN and TNMD expression after eAEC, mAEC, and tdAEC transplantation in injured tendons at day 28 post-surgery. In each panel the healthy portion (area 0 positioned on the left in all images) and the injured area of CTR and treated explants (from area 1 to area 5) are shown with white dashed lines. Representative images of (**A**) DCN and (**B**) TNMD positivity (green fluorescence) detected in the healthy portion of the explants (area 0) and within the injury site of eAEC-, mAEC-, and tdAEC-transplanted tendons. CTR tendons were negative for DCN, whereas TNMD was expressed in the cytoplasm of the cells contained within a disorganized ECM. In treated tendons both molecules at 28 days were expressed in an organized ECM. Nuclei were counterstained with DAPI. Scale bar = 100 µm. (**C**) Representative histograms of green fluorescence intensities of DCN and TNMD at 28 days CTR (black), eAEC- (green), mAEC- (light red), and tdAEC- treated (red) groups in all analyzed areas using RGB profiler plugin of ImageJ software (NIH). The green and red dashed lines represent the maximum and minimum green fluorescence intensity of DCN and TNMD, respectively. ^a^ Statistically significant vs. CTR (*p* < 0.05), ^b^ statistically significant vs. eAECs (*p* < 0.05), ^c^ statistically significant vs. mAECs (*p* < 0.05).

Similarly, the greater support on ECM remodeling induced by eAECs and tdAECs during the early phase of tendon healing seems to be confirmed also by the IHC and fluorescence intensity analyses of proteoglycan DCN deposition: in the totality of explants, DCN deposition displayed a uniform high degree of density with parallel fibers extended up to areas 4 and 5 (Figure 6A,C). On the contrary, DCN deposition in four out of five mAECs-treated tendons displayed a lower consistency that never exceeded area 3 (Figure 6A,C). Both treated tendons at day 14 (data not shown) and spontaneous tissue (CTR at both time points) did not express DCN (Figure 6A,C).

The allotransplantation of AEC subsets also affected the expression and distribution of the late tendon-related marker during the early stage of tendon healing. More in detail, TNMD became widespread in the whole injured tissue (from area 1 to area 5) in treated explants at day 28 (Figure 6B,C), whereas before the tendon-related protein displayed a faint positivity mainly localized in the core of the lesion (day 14: data not shown). Differently, the totality of CTR tendons (five out of five) were unable to assemble TNMD that resulted also after 28 days as a weak (Figure 6C) and disorganized positivity inside the injured zone (Figure 6B).

### 3.6. eAEC, mAEC, and tdAEC Allotransplantation Improves Cellularity and Cell Alignment in Injured Tendons

**Figure 7:**
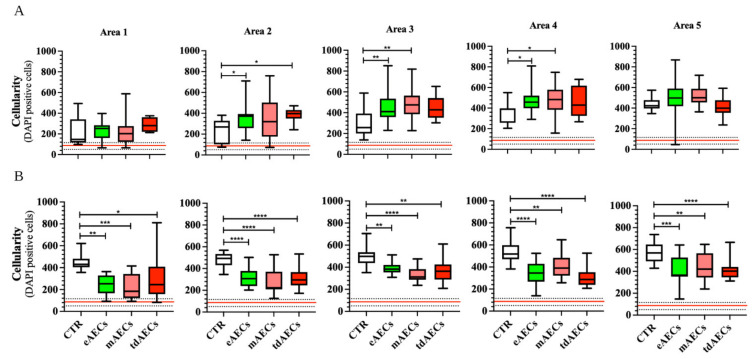
Spatial distribution of cellularity in healing tendons at day 14 and day 28. Box plots showing the number of cells within spontaneous healing tendons (CTR) and treated tendons with eAECs, mAECs, and tdAECs within 5 different analyzed fields in all 6 areas (from area 0 to area 5) after (**A**) 14 and (**B**) 28 days of tendon injury. Values were considered statistically significant for * (*p* < 0.05), ** (*p* < 0.01), *** (*p* < 0.001), and **** (*p* < 0.0001). The quantitative data were expressed as mean ± S.D.

**Figure 8:**
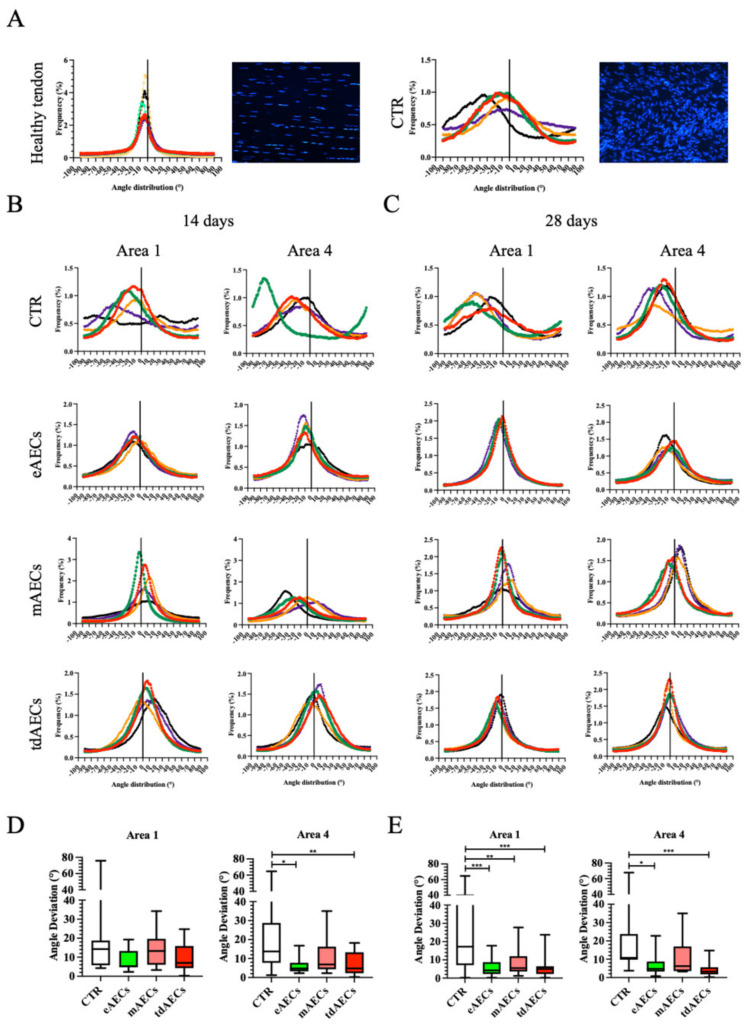
Directionality analyses on cell orientation of the different samples at 14 days and 28 days. (**A**) Representative directionality curves of the analyzed samples within healthy tendon area and CTR. Representative graphs showing the distribution of cell direction within area 1 and 4 in healing tendons under different conditions assessed through directionality of ImageJ at (**B**) 14 days and (**C**) 28 days. Histograms showing the angle deviation within healing tendons under different treatment conditions within area 1 and area 4 normalized to the healthy tendon area, used as reference for the analyses at (**D**) 14 days and (**E**) 28 days. Statistically significant values were set up for * *p* < 0.05, ** *p* < 0.01, and *** *p* < 0.0001. The quantitative data were expressed as mean ± S.D.

The morphometric analysis in terms of cellularity (Figure 7) and cell alignment (Figure 8) were performed by adopting a spatial approach starting from the healthy portion of the tendon (area 0 or red line in Figure 8) to move towards the core of the lesion (from area 1 to area 5), thus comparing six contiguous areas of 500 µm^2^ each, as previously described [7,30].

As expected, the lowest cellularity with an average of about 87 ± 27 cells/field was recorded in the healthy portion of the tendons (area 0; red line in Figure 7), whereas CTR and transplanted tendons showed a higher cellularity, even if cell treatment was able to affect the parameter in a time- and area-dependent manner. In detail, the data of cellularity were higher than CTR at day 14 in: areas 2, 3, and 4 of eAEC-transplanted tissues, in area 3 of mAEC-treated samples, and in area 2 of tdAEC ones (Figure 7A). On the contrary, a similar high cellularity was recorded in the core of the lesion (area 5), independent of tendon group (Figure 7A).

Of note, all subsets of AECs’ treatment induced a prompt widespread reduction in cell number that involved all the regenerating areas (areas 1–5) at day 28 (Figure 7B) by reaching values that were always significantly lower than those recorded in CTR injured zones (at least *p* < 0.05, Figure 7B). Especially in area 1 and 2, treated tendons exhibited a cellularity that was closer to that displayed by healthy tendons (Figure 7B). By approaching the core of the lesion site, eAECs and tdAECs became more efficient in modulating cellularity. Indeed, in both treatments induced in area 4 (mAECs, eAECs, and tdAECs, *p* < 0.001 and *p* < 0.0001, respectively) and area 5 (mAECs *p* < 0.01, eAECs *p* < 0.001, and tdAECs *p* < 0.0001) there was a significant reduction in cell number (Figure 7B).

The overall results on cellularity and ECM protein remodeling reinforced the evidence of AECs’ role in accelerating tendon healing, converging towards the hypothesis of a more targeted regenerative action of eAECs and tdAECs.

Moreover, in order to increase the information on tendon microarchitecture recovery, data of cell nucleus orientation were also collected by analyzing the distribution of cell direction (Figure 8). More in detail, data of angle deviation were compared with those of area 0, the positive control reproducing the values of cell direction in heathy tendons, and analyzed in comparison with areas 1 or 4, the two more representative zones of the spatial process of tendon regeneration (area 1 close to the healthy portion of tendon and area 4 close to the core of lesion). The distribution of cell orientation was characterized in healthy tendons by sharp Gaussian curves with an average of 5.25° ± 2.12 (Figure 8A). The analyses of angle distribution carried out within both area 1 and area 4 demonstrated that eAECs, mAECs, and tdAECs were able to positively influence tissue regeneration by significantly accelerating the phenomenon of cell orientation that did not occur during the early stage of spontaneous healing tissue (CTR). The process of cell alignment still improved at day 28 when the values and Gaussian curves became closer to those recorded in healthy tendons (Figure 8B,C).

In detail, the Gaussian curves of CTR tendons displayed a very high dispersion at 14 days after injury (Figure 8A, right corner), as clearly demonstrated by the comparison, first of all, with the curves’ shape of healthy tissues (Figure 8A, left corner: positive control), as well as with the treated tendons (Figure 8B), even if a clear AEC subsets’ effect on angle distribution was evident. Of note, only eAEC and tdAEC tendons displayed the Gaussian curves of both in area 1 and area 4, characterized by low levels of dispersion. On the contrary, a similar trend in cell angle distribution was obtained in mAEC-treated tendons exclusively in area 1 to then become broader by approaching area 4 (Figure 8B). Interestingly, the curves of directionality exhibited an overall (area 1 and area 4) remarkable enhancement in sharpness at 28 days and, in particular, for all AEC-treated tendons. Of interest, eAEC-treated tendons presented for area 1 the fitted Gaussian curves with a distribution similar to that analyzed in healthy tendons (Figure 8C).

Then, the parameter of angle deviation was analyzed by normalizing cell directions to those recorded in the cells belonging to healthy tendons (Figure 8D,E). The highest averages of angle deviation were recorded in CTR tissues at day 14. In comparison, all the treatments with AECs promoted a reduction in angle deviations and variabilities (Figure 8D) that became significantly lower in area 4 of eAEC- and tdAEC-treated tendons (CTR vs. eAECs: *p* < 0.05 and CTR vs. tdAECs: *p* < 0.01, Figure 8D).

The highest angle deviations and variability were still recorded in spontaneous healing tendons at day 28 (CTR, Figure 8E) when, on the contrary, all AEC subsets’ treatments promoted a relevant improvement in the angle deviation of the cells either close to the healthy portion of the tissue (area 1) or in proximity to the core of the lesion (area 4). More in detail, significantly lower angle deviations characterized area 1 of mAEC- (*p* < 0.01 vs. CTR), eAEC-, and tdAEC-treated tendons (vs. CTR for both *p* < 0.001) (Figure 8E). Otherwise, exclusively eAECs and tdAECs treatments were able to significantly enhance the angle deviation trend in area 4 (CTR vs. tdAECs, *p* < 0.001: CTR vs. eAECs, *p* < 0.05) (Figure 8E).

Overall, the quantitative analyses of cell alignment enlarged the findings, supporting the proof of concept of the enhancement of eAECs and tdAECs in the early stage of tendon regeneration.

### 3.7. AEC Subsets’ Direct Contribution to Tendon Regeneration by Differentiating into Tenocytes

Labelled PKH26 eAECs, mAECs, and tdAECs used for the allotransplantation were always retrieved within the engrafted tendons. As expected, the PKH26 fluorescent signal was retrieved over the cells and never in ECM.

**Figure 9:**
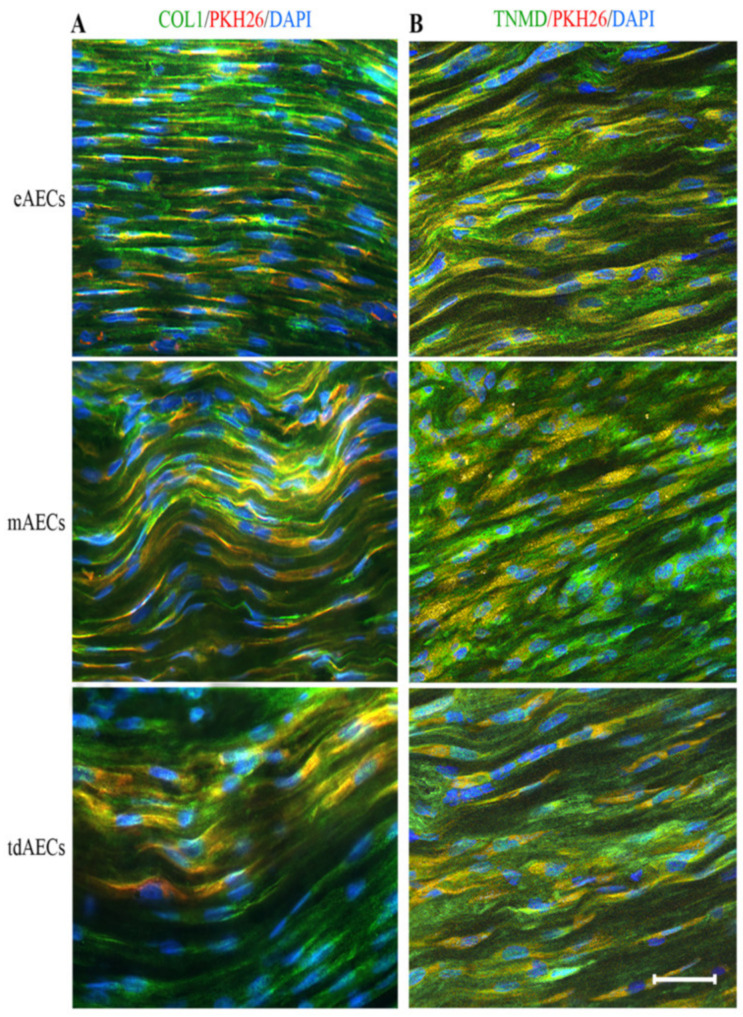
eAECs, mAECs, and tdAECs survived within the host tissue and differentiated into tenocytes. (**A**) Representative images captured at day 14 showing several PKH26-positive cells (red fluorescence) that co-localize with COL1 (green fluorescence), giving a yellow merged fluorescence. These AECs showed a fusiform shape and flattened nuclei (DAPI counterstaining). (**B**) Representative images obtained from samples at day 14 showing several PKH26-positive cells (red fluorescence) that co-localize with TNMD (green fluorescence), giving a yellow merged fluorescence. All three types of co-localizing AECs showed a fusiform shape and flattened nuclei (DAPI counterstaining). Scale bar = 50 µm.

PKH26-positive eAECs, mAECs, and tdAECs were entrapped within the ECM. The eAECs differentiated acquiring a fusiform shape already at 14 days after transplantation. In particular, most of eAEC–PKH26-labelled cells became, after in vivo transplantation, positive for COL1 (Figure 9A) and TNMD (Figure 9B), mainly accumulated into their cytoplasm. The fusiform shape persisted in both PKH26-labelled mAECs and tdAECs as well as their ability to synthetize COL1 (mAECs and tdAECs) and TNMD (tdAECs), as demonstrated by the yellow fluorescence that is the result of the merged red (PKH26) and green fluorescent signals of COL1 and TNMD (Figure 9A,B). Tenocyte-like PKH26-positive eAECs, mAECs, and tdAECs persisted also after 28 days transplantation (data not shown).

### 3.8. Inflammatory Phase Is Positively Modulated by eAEC, mAEC, and tdAEC Allotransplantation

**Figure 10:**
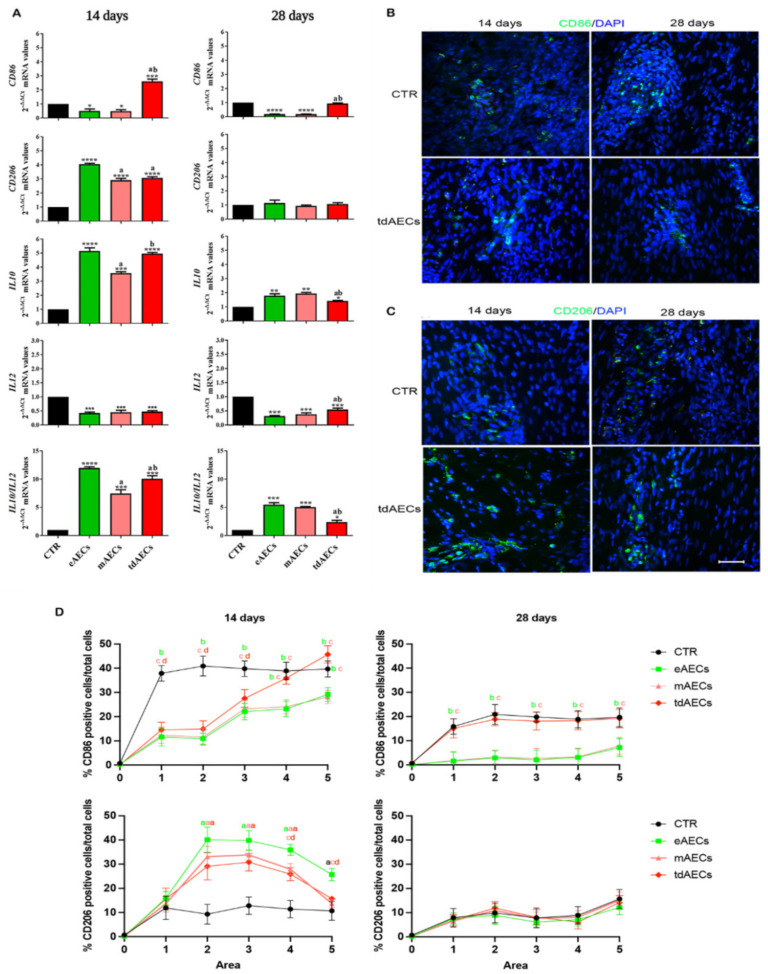
Gene expression and percentage of M1 and M2 macrophage markers and gene expression of interleukins *IL12* and *IL10* in explanted tendons. (**A**) Gene expression profile of M1 macrophage (*CD86*) and M2 macrophage (*CD206*) markers and pro-inflammatory *IL-12* and anti-inflammatory *IL-10* in CTR (black), eAEC- (green), mAEC- (light red) and tdAEC-treated (red) tendons at 14 and 28 days after cell transplantation. Statistically significant values were set up for * *p* < 0.05, ** *p* < 0.01, *** *p* < 0.001, and **** *p* < 0.0001 vs. CTR, ^a^ *p* < 0.05 vs. eAECs, and ^b^ *p* < 0.05 vs. mAECs. The quantitative data were expressed as mean ± S.D. (**B**) Representative images of CD86- and (**C**) CD206-positive cells (green fluorescence) in CTR and tdAEC-treated tendons, taken as an example, at day 14 and 28. Nuclei were counterstained with DAPI (blue fluorescence). Scale bar: 50 μm. (**D**) Quantification of CD86- and CD206-positive cells related to M1 and M2 macrophage phenotypes, respectively, in the defect area (from area 1 to area 5) of healing tendons performed at 14 and 28 days post-treatment. ^a^ Statistically significant vs. CTR (*p* < 0.05), ^b^ statistically significant vs. eAECs (*p* < 0.05), ^c^ statistically significant vs. mAECs (*p* < 0.05), and ^d^ statistically significant vs. tdAECs (*p* < 0.05).

In order to evaluate the evolution of the inflammatory phase in mechanically injured tendons, mRNA expression of *CD86* (pro-inflammatory M1Mφ phenotype), *CD206* (anti-inflammatory M2Mφ phenotype), and their relative major interleukins (*IL12* and *IL10*, respectively) were analyzed at 14 and 28 days (Figure 10A). In particular, all the AECs’ treatments induced at day 14 a significant upregulation of both anti-inflammatory gene markers, *CD206* and *IL10* (*p* < 0.0001 vs. CTR), with their highest levels in eAEC tendons (Figure 10A). Contextually, despite a common downregulation of *IL12* expression in all subsets of AEC-treated tendons, the M1Mφ transcript levels were strongly reduced exclusively in eAEC- and mAEC-transplanted tendons (Figure 10A), whereas tdAEC-treated samples displayed an upregulation of *CD86* that reached values that were significantly higher than CTR tissues (*p* < 0.001).

Of note, an overall higher IL10/IL12 characterized all subsets of AEC-treated tendons even if the more favorable ratio was recorded in eAEC-transplanted ones (Figure 10A).

An overall reduction in the pro- and anti-inflammatory gene profiles was observed after 28 days in treated tendons. As a consequence, the M1Mφ phenotype markers, *CD86*, reached levels of expression that were significantly lower than in CTR tissues in allotransplanted tendons with eAECs and mAECs or that were similar to CTR in tdAEC-transplanted ones (Figure 10A). Similarly, a lower influence on *IL2* expression was recorded after tdAEC transplantation even if AECs’ treatments, independently of the cell subsets, were always able to promote a pro-inflammatory cytokine profile that was significantly lower than in CTR (all AECs’ treatments vs. CTR: *p* < 0.001; Figure 10A). Moreover, despite CD206 mRNA level not differing amongst tendon groups (Figure 10A), *IL10* expression was significantly upregulated in the presence of all AEC subsets (CTR vs. eAECs and mAECs, *p* < 0.01 and CTR vs. tdAEC, *p* < 0.05; Figure 10). As consequence, a more favorable IL10/IL12 ratio was recorded in all tendons treated with AECs even if the ratio reached values of higher significance for eAECs and mAECs (vs. CTR, both *p* < 0.001) than for tdAECs’ treatment (vs. CTR, both *p* < 0.05; Figure 10A).

The presence and distribution of M1Mφ and M2Mφ subpopulations were evaluated within the lesion site by using CD86 and CD206 markers, respectively (Figure 10B,C). At day 14, the percentage of CD86-positive cells was lower in eAEC- and mAEC-treated tendons with respect to CTR and to tdAEC-treated tendons in area 4–5 (*p* < 0.05), where most of the M1Mφ were localized (Figure 10D). At day 28, the percentage of M1Mφ in eAEC- and mAEC-allotransplanted tendons further decreased, reaching values similar to those recorded into the healthy portion of the tendon. The percentage of CD86-positive cells in CTR and tdAEC tendons, instead, resulted still significantly high (*p* < 0.05; Figure 10D).

At day 14, the M2Mφ subpopulation was higher in allotransplanted tendons with respect to CTR tendons within areas 2, 3, and 4, and for eAECs also in area 5 (*p* < 0.05; Figure 10D). The eAEC-treated tendons showed significantly higher values with respect to mAECs and tdAECs in areas 4 and 5 (*p* < 0.05; Figure 10D). At day 28, the percentage of M2Mφ was significantly lower in all subsets of AEC-treated and CTR tendons (*p* > 0.05; Figure 10D).

**Figure 11:**
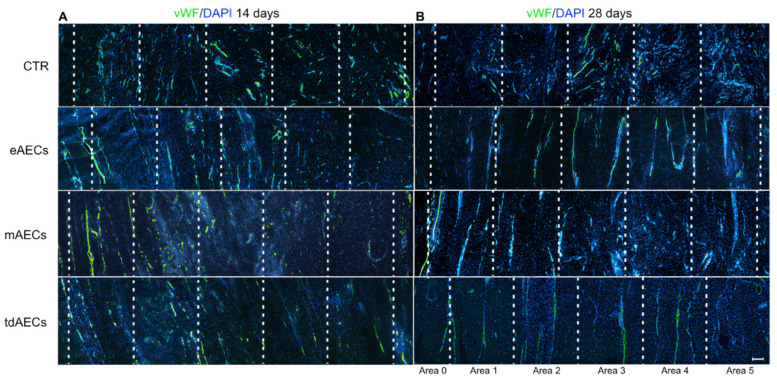
Tendon vascularization within CTR and treated tendons. Blood vessel distribution is documented in CTR and eAEC-, mAEC- and tdAEC-treated tendons at day 14 by immunohistochemistry using the endothelial marker, vWF (green fluorescence). Nuclei were counterstained with DAPI. In each panel the healthy portion (area 0 positioned on the left in all images) and the injured area of CTR and treated explants (from area 1 to area 5) are shown. Allotransplanted tendons displayed: (**A**) at day 14 the presence of blood vessels that were either scattered or aligned to the longitudinal axis of the tendon for an extension corresponding to maximum 1 area within the injured site and (**B**) at day 28, a limited number of blood vessels within the lesion; most of them ran parallel to the longitudinal axis of the tendon, similarly to those observed in the healthy portion of the explant, for an extension corresponding to up to 4 areas within the injured tendon. A more irregular organization was observed in the blood vessels localized in the core of the defect. (**A**,**B**) CTR tendons showed blood vessels irregularly distributed in the entire injury site. Scale bar = 100 µm.

Then, the effect of eAEC, mAEC, and tdAEC allotransplantation on inflammatory phase was investigated by analyzing blood vessel organization. In CTR tendons, independently of the time period of the explants (Figure 11A,B), blood vessels displayed a high vascular density and an irregular distribution inside the disorganized ECM of the injured area. A strong effect of all subsets of AECs’ transplantation was already visible at day 14 on the blood vessel remodeling (Figure 11A). A scattered blood vessel network was limited to area 1, whereas in the other areas of injured tissue aligned blood vessels oriented along the longitudinal axis of the tendon started to organize. Interestingly, at day 28, similarly to the healthy tendons, blood vessels appeared to be few and parallelly oriented (Figure 11B). As in previously morphological recovery gradient evidence, the blood vessels were completely rearranged at the periphery of the defect area, near the healthy tendon (area 1), whereas in the core of the lesion (area 5) the observation of an irregular scattered network was more frequent (Figure 11B). In addition, this qualitative analysis showed that eAEC- and tdAEC-treated tendons have developed an advanced process of blood vessel remodeling with regular parallel blood vessels covering most of the injured zone (from area 1 up to area 4), while such a morphological detection was limited in the mAEC-treated tendon where an oriented parallel blood vessel network never went beyond area 3 (Figure 11B).

### 3.9. Allotransplantation Modified the Response of the Injured Host Tissue: The Total Histological Score Assessment

**Figure 12:**
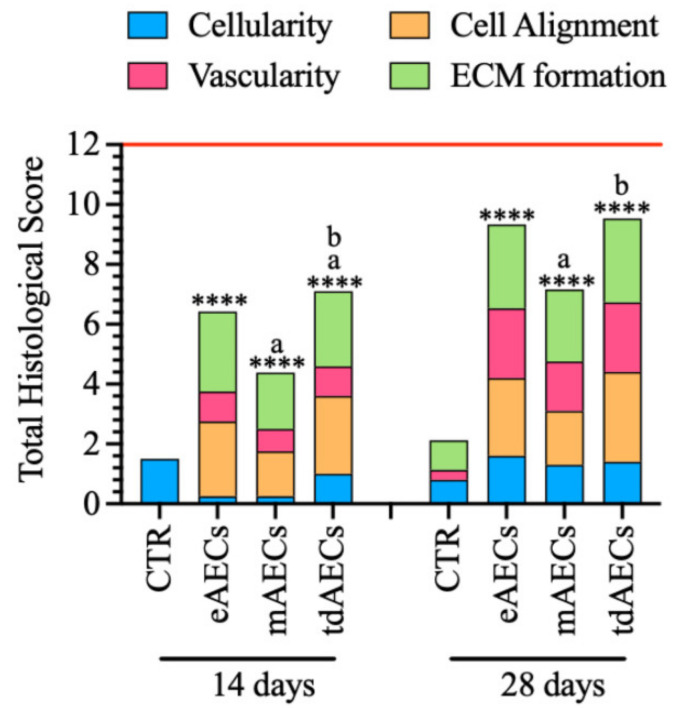
Stacked histograms of the semiquantitative histomorphometric score for stem-cell-treated and CTR groups at 14 and 28 days. The total histological score was compared to healthy tendon (red line) and calculated in terms of cellularity, cell alignment, vascularity, and ECM formation and structure scores (Mean). Statistically significant values were set up for **** vs. CTR (*p* < 0.0001) and ^a^ vs. eAECs (*p* < 0.0001) and ^b^ vs. mAECs (*p* < 0.0001). The quantitative data were expressed as mean ± S.D.

An overall assessment of early tendon healing due to eAEC, mAEC, and tdAEC transplantation was compared after 14 and 28 days by summarizing the obtained histomorphometric results according to the scores and sub-scores described in Table 3. As shown in Figure 12, the total histological scores (THS) assigned to different tendon groups displayed a significantly regenerative enhancement induced by all subsets of AEC treatments at both 14 and 28 days (*p* < 0.0001). The highest qualitative THS values was, however, achieved by tdAECs at 14 days and by tdAECs and eAECs at 28 days (Figure 12), even if they were both far from reaching the maximum score of 12 that characterized the healthy tendons (red line in Figure 12).

On the other hand, even if all subsets of transplanted cells greatly influenced the modulation of THS in the host tissue, the sub-scores were largely affected by the different cell subsets and by the timing of tissue regeneration.

In detail, the sub-scores in the CTR tendons were always lower than in treated groups, except for cellularity at 14 days (Figure 12). Concerning the average cellularity, at day 14 tdAECs (sub-score 1) showed the highest value (mAECs and eAECs sub-scores 0.25), whereas at 28 days the reduction in cellularity was better supported by eAECs (eAECs 1.6, mAECs 1.3, tdAECs 1.4). Cell alignment showed an increasing improvement in area 4, the nearest area to the core of the lesion, between 14 and 28 days, with the highest score for tdAEC-treated (sub-scores 2.5 and 3) followed by eAEC- (sub-scores 2.5 and 2.6) and mAEC-treated samples (sub-scores 1.5 and 1.8), respectively. Moreover, fiber organization in terms of COL1 expression showed at 14 days the highest sub-score for eAEC-treated (sub-score 2.67) followed by tdAEC- (sub-score 2.5) and mAEC-treated (sub-score 1.88) tendons. These sub-scores increased after 28 days, with highest values attributed to both eAEC and tdAEC (sub-scores 2.8) followed by mAEC (sub-score 2.4) samples. Finally, the vascularity showed a progressive amelioration of the blood vessel distribution within the injured area, in which eAEC- and tdAEC-treated tendons showed similar values of 1 and 2.33 at 14 and 28 days, respectively. The mAEC-treated tendons, instead, had lower sub-score values compared to eAECs and tdAECs both at day 14 (sub-score 0.75) and day 28 (sub-score 1.75).

## 4. Discussion

In this research, for the first time, the in vivo regenerative potential of the three different cell subsets (i.e., eAECs, mAECs, and tdAECs) obtained under specific in vitro culture conditions from the same naïve AEC stem cell source on a validated and highly translation value ovine Achilles tendon injury model was compared simultaneously. Despite the defect not being considered critical, the regenerative mechanisms induced by transplanted cells in the host tissue have been proven via in-depth study [17,18].

The conducted in vivo experiments have demonstrated that, with respect to tendon spontaneous healing, all three AEC subsets used were able to modulate tendon and ECM-specific markers, enhancing the regenerative process already after 14 days post-injury. Even if mAEC-transplanted tendons showed an overall upregulation of the tenogenic marker expression, with respect to CTR, these were less performant compared to eAEC- and tdAEC-treated tendons. The tdAEC-treated tendons promptly upregulated *COL1* and *THSB4*, but most of all in the host tissue they accelerated the substitution of COL3 with COL1. Similar results were obtained by eAEC transplantation, that in the host tissue, taking advantage of their great plasticity, they were able to quickly trans-differentiate by acquiring a tenocyte-like phenotype expressing COL1 and TNMD. This evidence is in accordance with the in vivo stepwise trans-differentiation towards the tenogenic lineage of hAECs within the ovine host tissue [17]. This pro-regenerative environment promoted by eAEC transplantation may be responsible for the highest expression of the tendon-related genes *SCX* and *TNMD* in the host tissue. SCX has a key role in driving the tendon-regenerative influence of eAECs, since this transcription factor is involved in the regulation of TNMD by addressing tendons towards a more mature tissue [31,32,33,34]. In addition, the early phase of tendon healing was supported by the different AEC subsets through the upregulation of *TGF-β1*. Of note, its signaling is essential for the maintenance of tendon cell fate [35] and collagen protein synthesis [36] by increasing the production of COL1 and COL3 [37]. Accordingly, in the present research there was an induction of COL1 protein expression in all allotransplanted tendons, suggesting that the engrafted cells, directly or through a mediated action of growth factors and local progenitor cells, were able to stimulate a greater deposition of collagen fibers [38].

The IHC analysis showed that, differently to CTR, treated tendons displayed the first signs of tendon microarchitecture recovery with COL3 immature fibers that at 14 days started to be replaced by a COL1 mature form of collagen that accounts for more than 90% of the ECM in healthy tendons [38]. However, at this time point collagen fiber deposition was strictly dependent on cell subset. In fact, eAEC-treated tendons showed a more homogeneous COL1 deposition pattern compared to mAEC- and tdAEC-treated tendons, which had parallelly deposited COL1 fibers but alternated with fainter deposition areas. It is conceivable that eAECs produced a better collagenous aligned ECM upon transplantation into injured Achilles tendons, since at day 14 they expressed higher mRNA levels of *SCX* and *TNMD* required for proper COL1 matrix assembly and structural integrity [39], as well as prevention of fibrovascular scar formation during early tendon healing [40].

Moreover, after 14 days, DCN and TNMD protein expression were barely detectable in all samples, even if TNMD positivity was visible in the core of the lesion, where PKH26-positive cells were mainly found. The mean cell number within the eAEC-, mAEC-, and tdAEC-treated tendons was higher with respect to CTR, which is a typical characteristic of the tendon healing proliferative phase [37]. In addition, cell directionality analyses were significantly higher for eAECs and tdAECs in area 4, near the core of the lesion with respect to CTR. Overall, these results demonstrate the ongoing ECM remodeling process that is consolidated at 28 days after transplantation. In particular, at this time point the expression of TGF-β1 and the tendon-related genes was overall downregulated, except for *COL1* which maintained its expression on high values by supporting a more favorable *COL1*:*COL3* ratio than in CTR tendons. TGF-β1 downregulation can be positively considered, since its modulation is necessary to avoid fibrotic scar tissue formation [41,42,43] and address a correct regenerative process. This supports an anticipated maturation regenerative phase, justified by the downregulation of the tendon-related genes, except for *COL1* which still must complete the replacement and rebuilding of the ECM present in the injured sites. This hypothesis is confirmed by the evidence that at 28 days after transplantation the host tissue was characterized by a decreased cellularity, showing high levels of COL1 deposition and density with a regular pattern completely replacing COL3, which represents an indicator of the tendon repair process [44]. Indeed, the replacement of COL3 immature fibers by COL1 is required to generate the mature form of ECM characterized by higher biomechanical properties [17,45]. Actually, the reduced strength of the repaired tissue results from reduced integration of collagen fibers and from a higher ratio of COL3 with respect to COL1 [37]. COL3 contributes to tensile stress by decreasing the elasticity and increasing the weakness of collagen tissues [45,46,47]. The ECM maturation process was also confirmed by the expression of DCN and TNMD within the repairing sites. DCN is the most abundant proteoglycan in tendons and has an important role in fibrillogenesis during development and maturation [48]. Moreover, it also influences the mechanical properties of tendons, transferring the load to collagen fibrils and promoting slides between fibrils [49].

The regular deposition of the ECM during matrix maturation significantly decreased cell density and was accompanied by the adoption of cell alignment by the matured tenocytes, especially in eAEC- and tdAEC-treated tendons with respect to mAEC samples. In particular, it was observed that in the majority of eAEC- and tdAEC-treated tissues there was a high density and regularly deposited COL1 for a higher extension with respect to mAEC-transplanted tissues, showing thus a slight delay in tendon recovery with respect to eAEC and tdAEC transplantation. The more effective influence of eAECs and tdAECs to the proliferative phase of tendon healing seems to be confirmed also by the improved distribution of somatic cell angle direction and deviation. Indeed, at day 28, these in CTR groups maintained high variability, whereas in treated tendons, especially eAEC and tdAEC ones, they displayed a remarkable curve sharpness. Altogether, these results strongly demonstrate that all transplanted AECs, and especially eAECs and tdAECs, accelerated tendon regeneration through a positive process of early COL1, TNMD, and DCN synthesis and protein deposition and maturation.

The crosstalk between eAECs, mAECs, and tdAECs and the host tendon also involved other cellular districts, as demonstrated by the analyses of local modulation/activation of inflammatory mechanisms and blood vessel remodeling. All three types of transplanted AECs promoted a prompt reorganization of the blood vessel network in the defect area already after 14 days post-surgery, even if a lower density and the realignment toward the longitudinal axis of the healthy tendon was reached by blood vessels only at day 28. The modulation of the angiogenic process could have a double positive effect on tendon healing: on one hand, it depicts a key element of homeostasis restoration, and, on the other hand, it strongly contributes to define the biomechanical properties of ECM. Active angiogenesis is required for a rapid formation of the intravascular plexus during the granulation tissue development that occurs immediately after an injury. However, the complete recovery of tendon biomechanical properties does not occur until a specific blood vessel network replaces the vascular plexus [50]. In this context, eAECs, mAECs, and tdAECs also induced tendon healing through a rapid blood vessel regression and remodeling that promoted the regenerative program.

In order to provide insights into the mechanisms involved in tendon ECM regeneration, the comparison of cellularity, cell alignment, COL1 deposition, and blood vessel remodeling deserves further attention. All these parameters, especially at day 28, exclusively in transplanted tissues, followed a gradient distribution (from the healthy tendon to the core of the defect area), confirming that AECs were able to promote a centripetal regenerative process [17,18]. Therefore, all three subsets of AECs through accurate paracrine actions were able to modulate an orderly process of collagen deposition and blood vessel reorganization [51,52].

Moreover, the enhanced modulation of the inflammatory phase promoted by the allotransplanted eAECs, mAECs, and tdAECs represented another relevant positive event leading to the early tendon healing. First of all, as recently demonstrated in vivo also for oAECs and hAECs [17,18,19], transplantation modulated the innate immune response of host tissue by promoting the inhibition of the pro-inflammatory M1Mφ and the parallel activation of the anti-inflammatory/pro-regenerative M2Mφ subpopulation. In particular, after 14 days in both eAEC- and mAEC-transplanted tendons, the pro-inflammatory *CD86* mRNA was strongly downregulated and anti-inflammatory *CD206* upregulated, confirmed also by their protein expression especially in eAEC-treated tendons, with respect to CTR, while tdAEC samples showed higher values for both *CD86* and *CD206,* showing a sort of balancing between these two markers. In accordance with the cell type, there was a significant downregulation of *IL12* mRNA expression and a significant upregulation of *IL10* mRNA expression with particular regard to eAECs and tdAECs, with a consequent *IL10/IL12* ratio that was higher in eAECs with respect to mAECs- and tdAECs- transplanted tendons. The increased local expression of M2Mφ-associated genes and proteins and the high *IL10/IL12* ratio recorded in the presence of all AEC subsets is in line with a rapid transition from the inflammatory to the reparative phase [53]. Moreover, this suggested an Mφ skewing towards an M2 phenotype, along with simultaneous higher expression of IL10, which is essential to prevent the production of pro-inflammatory cells/factors [54,55,56,57]. After 28 days, CD86 and CD206 mRNA and protein expression was downregulated in all transplanted tissues, as for *IL12* mRNA and *IL10* mRNA levels that were lower than those of 14 days. However, the *IL10/IL12* ratio remained higher in eAECs and mAECs with respect to tdAEC-transplanted tendons. The cytokine expression profile was specular in CTR: here, the prevalence of pro-inflammatory M1Mφ markers (CD68) and the local presence of ongoing inflammatory molecules (high concentration of IL12 mRNA) persisted over 28 days, probably leading to a spontaneous fibrotic scar evolution [19].

Indeed, it was confirmed in this in vivo model that eAECs possessed enhanced immunomodulatory properties with respect to mAECs or tdAECs.

Overall, the effect of eAEC, mAEC, and tdAEC transplantation on tendon regeneration vs. CTR can be summarized with the THS, which allowed also to quantify and compare the regenerative performances of the transplanted cells’ subsets. From this analysis, it was evident that treated groups always had significantly higher values with respect to CTR. The highest THS at day 14 was attributed to tdAECs that showed significantly different values with respect to eAECs and mAECs, whereas at day 28 eAECs and tdAECs were both significant with respect to mAEC-treated tendons, confirming their repairing delay. It must be considered though that the THS does not consider the immunomodulatory-related markers, which were much more favorable in eAECs in terms of M2Mφ marker and IL10 expression, giving them a great advantage over the other two cell types. Moreover, eAECs’ higher immunomodulatory attitude has probably privileged this cell type over mAECs in regenerating the injured tendon, whereas tdAECs were already committed and predisposed to teno-regenerate helped by, even if to a lesser extent with respect to eAECs, their preserved immunomodulatory capabilities.

However, before considering these cells applicable in human or vet clinical trials, further studies must be carried out on critical defects for tendons and eventually contemplate using their secretomes to overcome cell-based therapy limitations in humans. Furthermore, even if this research has analyzed in depth several regenerative mechanisms during tendon healing, a comprehensive determination of the underlying tenogenic regenerative mechanisms remains elusive. Thus, further investigations may be important to better understand if novel putative EMT and immunomodulatory regulators may represent new treatment strategies.

## 5. Conclusions

In conclusion, the present paper represents the first proof of concept that all three subsets of produced cells (i.e., eAECs, mAECs, and tdAECs) are able to accelerate tendon regeneration, providing therapeutic benefits during acute tendon healing and thus reinforcing the evidence of their role in regenerative medicine. However, mAECs showed reduced in vivo performances in terms of ECM remodeling with respect to eAECs and tdAECs. These latter, in particular, displayed two different underlying regenerative mechanisms. The eAECs positively influenced regeneration, mainly modulating the pro-inflammatory to pro-regenerative responses of the injured host tissue. This led, as a consequence, to an acceleration of the restoration of tendon microarchitecture. On the other hand, tdAEC transplantation supported a prompt recovery of the ECM, reducing cellularity and efficiently ameliorating fiber alignment of host cell compartment and reorganizing tendon microarchitecture. However, tdAECs exhibited a lower performance in immunomodulating the host response with respect to the other two cell subsets.

Thus, the obtained evidence suggests that eAECs, due to their greater plasticity and strong immunomodulatory properties, are a practicable and efficient strategy for treatment of acute tendinopathies, which are characterized by an extensive inflammatory process. P_4_ culture strategy could represent a critical standardization able to preserve the native biological and functional characteristics of AECs.

Thus, the obtained data encourage moving towards translation of this stem cell source to clinical practice in veterinary and human patients.

## Figures and Tables

**Table 1:** Primers details used for RT-qPCR analysis.

	Gene	Forward	Reverse
EMT	*VIM* ^a^	5′-GACCAGCTCACCAACGACA-3′	5′-CTCCTCCTGCAACTTCTCCC-3′
	*CYTO8* ^b^	5′-CTCAAAGGCCAGAGGGCTTC-3′	5′-CTTGGCCTGAGCATCCTTGA-3
	*SNAIL* ^a^	5′-GTCGTGGGTGGAGAGCTTTG-3′	5′-TGCTGGAAAGTGAGCTCTGG-3′
	*TWIST* ^a^	5′-GCCGGAGACCTAGATGTCATTG-3′	5′-CCACGCCCTGTTTCTTTGAAT-3′
TENOGENIC	*TNMD* ^a^	5′-TGGTGAAGACCTTCACTTTCC-3′	5′-TTAAACCCTCCCCAGCATGC-3′
	*SCXB* ^a^	5′-AACAGCGTGAACACGGCTTTC-3′	5′-TTTCTCTGGTTGCTGAGGCAG-3′
	*COL1* ^a^	5′-CGTGATCTGCGACGAACTTAA-3′	5′-GTCCAGGAAGTCCAGGTTGT-3′
	*COL3* ^c,d^	5′-AAGGGCAGGGAACAACTTGAT-3′	5′-GTGGGCAAACTGCACAACATT-3′
	*TBSH4* ^c,d^	5′-CCGCAGGTCTTTGACCTTCT-3′	5′-CAGGTAACGGAGGATGGCTTT-3′
IMMUNO	*CD86* ^c,d^	5′-AGAAGGTCCCAAGGACTGGT-3′	5′-GCTTGGCACAGGTGACTTTG-3′
	*CD206* ^c,d^	5′-GTAGAAGCAGGCTGCCAGAA-3′	5′-CTTCTGCCCAGTGTTTGCAC-3′
	*IL10* ^c,d^	5′-CTGTGCCTCTCCCCTAGAGT-3′	5′-GCAGCTAGCTCCACAAGGAA-3′
	*1L12* ^c,d^	5′-ACAAAGGAGGCGAGGTTCTG-3′	5′-CTGTGGTCCATGCTGACCTT-3′
H.K.	*GAPDH* ^a^	5′-CCTGCACCACCAACTGCTTG-3′	5′-TTGAGCTCAGGGATGACCTTG-3′

Primers used in previous reports: ^a^ [7], ^b^ [9], ^c^ [17], ^d^ [19]. Legend. EMT: epithelial mesenchymal transition genes, tenogenic: tendon-related genes, immuno: immunomodulatory genes, H.K: housekeeping gene.

**Table 2:** Primary and secondary antibodies used for ICC and tendon analysis.

	Primary Antibody	Dilution	Secondary Antibody	Dilution
EMT	CYTO8 (Abcam, Cambridge, UK)	1:200	Anti-mouse Alexa Fluor (Sigma-Aldrich, St. Louis, MO, USA)	1:500
	VIM (Agilent Technologies, Santa Clara, CA, USA)	1:200	Anti-mouse Cy3 (Sigma-Aldrich, St. Louis, MO, USA)	1:750
TENDON MARKERS	COL1 (Chemicon Int., Billrerica, MA, USA)	1:200	Anti-Mouse Alexa Fluor 488 (Invitrogen Ltd., Paisley, UK)	1:400
	COL3 (Chemicon Int., Billrerica, MA, USA)	1:500	Anti-Mouse Alexa Fluor 488 (Invitrogen Ltd., Paisley, UK)	1:400
	TNMD (Abacm, Cambridge, UK)	1:100	Anti-Rabbit Alexa Fluor 488 (Invitrogen Ltd., Paisley, UK)	1:400
	DCN (Invitrogen, Waltham, MA, USA)	1:100	Anti-Rabbit Alexa Fluor 488 (Invitrogen Ltd., Paisley, UK)	1:400
IMMUNE MARKERS	CD86 (AbD serotec, a Bio-Rad Company, Hercules, CA, USA)	1:50	Anti-Mouse Alexa Fluor 488 (Invitrogen Ltd., Paisley, UK)	1:200
	CD206 (RδD Systems, a Bio-techne brand, 614 McKinley Place NE Minneapolis, MN 55,413 USA)	1:25	Anti-Goat Alexa Fluor 488 (Molecular Probes, Eugene, OR, USA)	1:200
	vWF (Dako Cytomation, Denmark)	1:400	Alexa Fluor 488 Anti-Rabbit (Invitrogen Ltd., Paisley, UK)	1:400

**Table 3:** Semiquantitative histomorphometric score.

Parameter	Score 0	Score 1	Score 2	Score 3
Cellularity expressed as average of cell N° in CTR and treated samples within all analyzed areas/average of cell N° in healthy tendons	Fold change > 4.5	4.5 < fold change > 3.1	3 < fold change > 1.5	Fold change < 1.5
Cell nuclei alignment expressed as the ratio between angle distribution of CTR and treated groups vs. healthy tendons along the longitudinal axis of the tendon within area 4	Ratio > 3 corresponding to an irregular cell distribution and alignment along the longitudinal axis of the tendon	3 < Ratio > 2.1 corresponding to cells that start to acquire a parallel orientation to the longitudinal axis of the tendon with high variability	2 < Ratio > 1 corresponding to cells that start to acquire a parallel orientation to the longitudinal axis of the tendon with low variability	Ratio < 1 corresponding to cells that acquire a parallel orientation to the longitudinal axis of the tendon
Vascularity	Vascular plexus	Presence of blood vessels that are either scattered or aligned to the longitudinal axis of the tendon for an extension corresponding to max 1 area within the injured tendon	Blood vessels aligned to the longitudinal axis of the tendon for an extension corresponding to 1 up to 4 areas within the injured tendon	Blood vessels oriented along the longitudinal axis of the tendon for an extension corresponding to up to 5 areas within the injured tendon
Fiber organization of COL1 within ECM	No COL1 expression and no fibers’ organization within the injured tendon	COL1 expression with fiber formation with an irregular distribution within the injured tendon	COL1 expression with scattered aligned fibers noted only for a small extension within the injured tendon	COL1 expression with aligned fibers noted for a wide extension within the injured tendon

## Data Availability

The data supporting reported results can be available upon request to the authors.
